# Development of Gallic Acid-Modified Hydrogels Using Interpenetrating Chitosan Network and Evaluation of Their Antioxidant Activity

**DOI:** 10.3390/molecules22111976

**Published:** 2017-11-15

**Authors:** Byungman Kang, Temmy Pegarro Vales, Byoung-Ki Cho, Jong-Ki Kim, Ho-Joong Kim

**Affiliations:** 1 Nuclear Chemistry Research Division, Korea Atomic Energy Research Institute, 989-111 Daedeok-daero, Yuseong-gu, Daejeon 34057, Korea; alchem95@gmail.com; 2 Department of Chemistry, Chosun University, Gwangju 61452, Korea; valestemmy@gmail.com; 3 Department of Natural Sciences, Caraga State University, Butuan City 8600, Philippines; 4 Department of Chemistry, Dankook University, 119, Dandae-ro, Chungnam 31116, Korea; chobk@dankook.ac.kr; 5 Department of Biomedical Engineering, School of Medicine, Catholic University of Daegu, Daegu 42472, Korea

**Keywords:** chitosan, IPN, hydrogels, antioxidant activity, gallic acid

## Abstract

In this work, antioxidant hydrogels were prepared by the construction of an interpenetrating chitosan network and functionalization with gallic acid. The poly(2-hydroxyethyl methacrylate) p(HEMA)-based hydrogels were first synthesized and subsequently surface-modified with an interpenetrating polymer network (IPN) structure prepared with methacrylamide chitosan via free radical polymerization. The resulting chitosan-IPN hydrogels were surface-functionalized with gallic acid through an amide coupling reaction, which afforded the antioxidant hydrogels. Notably, gallic-acid-modified hydrogels based on a longer chitosan backbone exhibited superior antioxidant activity than their counterpart with a shorter chitosan moiety; this correlated to the amount of gallic acid attached to the chitosan backbone. Moreover, the surface contact angles of the chitosan-modified hydrogels decreased, indicating that surface functionalization of the hydrogels with chitosan-IPN increased the wettability because of the presence of the hydrophilic chitosan network chain. Our study indicates that chitosan-IPN hydrogels may facilitate the development of applications in biomedical devices and ophthalmic materials.

## 1. Introduction

Biomaterials with unprecedented levels of structural organization and extraordinary properties have been sought for many years. One emerging material for the design and synthesis of functional biomaterials is hydrogels. Hydrogels are robust, three-dimensional cross-linked hydrophilic polymeric materials, which are capable of retaining large amounts of water and biological fluids [1,2,3]. More than 50 years after their discovery, poly(2-hydroxyethyl methacrylate) p(HEMA)-based hydrogels remain a prominent and relevant member of the hydrogel family. Owing to their intrinsic high biocompatibility, good mechanical properties, and excellent swelling behavior, p(HEMA)-based hydrogels are employed as biomedical materials, such as in drug delivery systems [4,5,6], contact lenses [7], dental adhesives [8], and carrier materials for wound healing [9,10]. In the past few decades, there has been a shift toward multi-component hydrogels. Some strategies for the preparation of multi-component hydrogels include thiol-ene/yne click reactions [11,12], native chemical ligation [13,14], oxime chemistry [15], and interpenetrating polymer networks (IPNs) [16]. In particular, an IPN is an intriguing system comprising cross-linked polymers, with at least one being synthesized and/or cross-linked within the immediate presence of the other. In an IPN system, the polymer networks are physically interlocked and entangled on the molecular scale without covalent bonds between the different types of polymer chains [17,18,19]. Furthermore, the fabrication of hydrogels via IPNs is typically simple and straightforward, where pre-polymerized hydrogels are submerged into a solution of monomers in the presence of a polymerization initiator. The resulting double network structure generally produces an advanced multicomponent hybrid system, which often exhibits significantly improved component polymer properties [20,21,22] and synergistic properties of the constituent polymers [23].

In recent years, advances in polymer science and biotechnology have facilitated the production of biomaterials with excellent bioactivities. Particularly, antioxidants have gained considerable attention in biomedical applications because of their ability to act as reducing agents, hydrogen-donating antioxidants, free radical scavengers, and single oxygen quenchers [24]. An important class of antioxidants is polyphenols, which have a distinguished ability to undergo oxidation/reduction reactions. For instance, gallic acid and caffeic acid play key roles in the defense mechanism against free radicals and reactive oxygen species (ROS) by breaking the free radical chain reaction via the hydroxyl groups on their aromatic rings [25,26]. However, the use of bare antioxidants in the pharmaceutical, biomedical, and food industries has faced various challenges, such as volatilization, instability, and oxidation under ambient oxygen [27,28]. Hence, antioxidants have been functionalized into biopolymers and inorganic materials to address the aforementioned impediments. Moreover, by using the advantages of each constituent, antioxidant–biopolymer conjugates could be employed as new food additives, in packing, and as biomedical materials. Several studies have reported on the functionalization of biomacromolecules such as chitosan and its derivatives with phenolic compounds extracted from plants, such as gallic acid, caffeic acid, tannic acid, and catechin [29,30,31,32]. Furthermore, the antioxidant curcumin has been incorporated into bandages and collagen matrices to promote wound healing [33].

**Figure 1:**
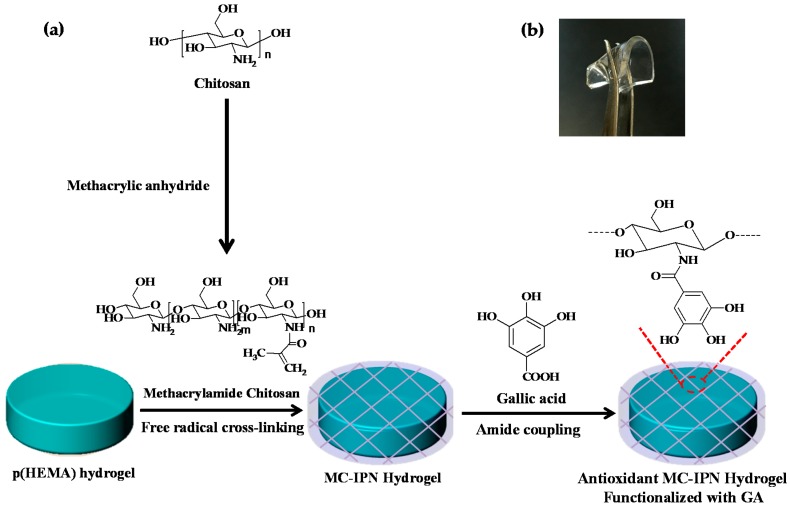
(**a**) Schematic representation for development of chitosan-interpenetrating polymer network (IPN) hydrogels functionalized with polyphenols; (**b**) Photograph of the fabricated antioxidant hydrogel.

Recently, we demonstrated the potential of IPN structures consisting of succinyl chitosan polymers and spiropyran as photochromic hydrogels [34]. The nucleophilic amino groups of chitosan polymers within the IPN structure facilitated modifications and conjugations with various functional molecules. In the present paper, we report a synthetic strategy for the preparation of antioxidant hydrogels using a simple method by constructing an IPN architecture based on methacrylamide chitosan (MC) and an antioxidant polyphenol, gallic acid (Figure 1). Initially, p(HEMA)-based hydrogels were synthesized and a chitosan-IPN was constructed using intermolecularly cross-linked chitosan chains and p(HEMA) networks. By means of amide coupling reactions, chitosan-IPN hydrogels were further surface-functionalized with gallic acid, which significantly improved the antioxidant activity of the hydrogels. The radical scavenging efficiency of the fabricated antioxidant hydrogels was investigated in two model assays employing 2,2-diphenyl-1-picrylhydrazyl (DPPH) and 3-ethylbenzothiazoline-6-sulfonic acid (ABTS) free radicals.

## 2. Results and Discussion

**Figure 2:**
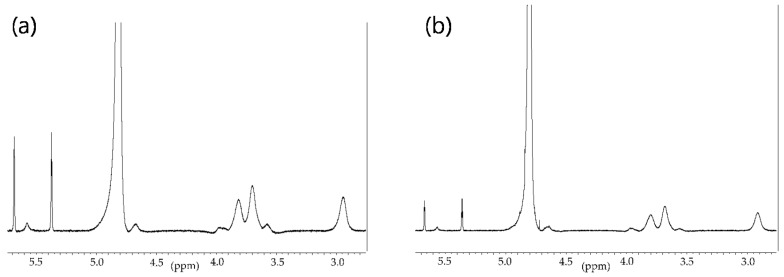
^1^H-NMR spectra of (**a**) low-methacrylamide chitosan (MC) gallic acid (GA); and (**b**) high-MC-GA.

Antioxidant hydrogels were prepared according to the synthetic route illustrated in Figure 1. Initially, p(HEMA)-based hydrogels were synthesized via free radical polymerization with HEMA monomers using ethylene glycol dimethacrylate (EGDMA) as a cross-linking agent and azobisisobutyronitrile (AIBN) as the initiator. The resulting p(HEMA)-based hydrogels were surface-modified with an IPN structure using cross-linked chitosan chains. Chitosan is a natural polysaccharide that has been used in various biomedical applications because of its biodegradability, nontoxicity, and biocompatibility. However, chitosan has limited solubility in both water and common organic solvents because of extensive intramolecular and intermolecular hydrogen bonding in the α- and β-conformations [35,36]. Although chitosans with an acetylation degree in the range of 40–60% and a medium molecular weight are soluble at physiological pH values [37], they must be chemically modified to improve the solubility in neutral aqueous media and common organic solvents and to be processed into IPN hydrogels. Herein, chitosan polymers were chemically modified by introducing methacrylate functionalities onto the *N*-position of the primary amine groups in the chitosan backbone, yielding a methacrylamide derivative of chitosan (MC). MCs with different molecular weights (MWs) were synthesized using 100–300 kDa and 600–800 kDa chitosan, for low- and high-MC, respectively. ^1^H-NMR spectroscopic measurements (Figure 2) revealed the degrees of methacrylation, which were found to be about 59.30% and 37.78% for low- and high-MC, respectively. The degree of methacrylation was calculated according to previously reported literature [38] by comparing the integrated area of the H2–H6 peaks at 2.8–4.0 ppm to that of the methylene peaks at 5.35 and 5.65 ppm.

Next, chitosan-IPN hydrogels (low-MC-H and high-MC-H based on low-MC and high-MC, respectively) were constructed by loading MC into p(HEMA) hydrogels, followed by the cross-linking of MC via radical polymerization across the methacryl carbon–carbon double bond using ammonium persulfate (APS) and sodium metabisulfite (SMBS) as polymerization initiators. The fabricated chitosan-IPN hydrogels were surface-functionalized by amide coupling reactions of antioxidant gallic acid (GA) to the chitosan network, which resulted in two antioxidant hydrogels, low-MC-GA and high-MC-GA based on low- and high-MC-H, respectively. After the cross-linking reaction, the yields of surface modification with chitosans were estimated to be ~61.6% and ~73.8% for low- and high-MC-H, respectively. The amounts of conjugated MC corresponded to about 0.123 and 0.148 g for low- and high-MC-H, respectively, for 1 g of the corresponding hydrogels. This was obtained simply by measuring the amount of MC bound after the cross-linking reaction and comparing it with the initial amount of MC.

**Figure 3:**
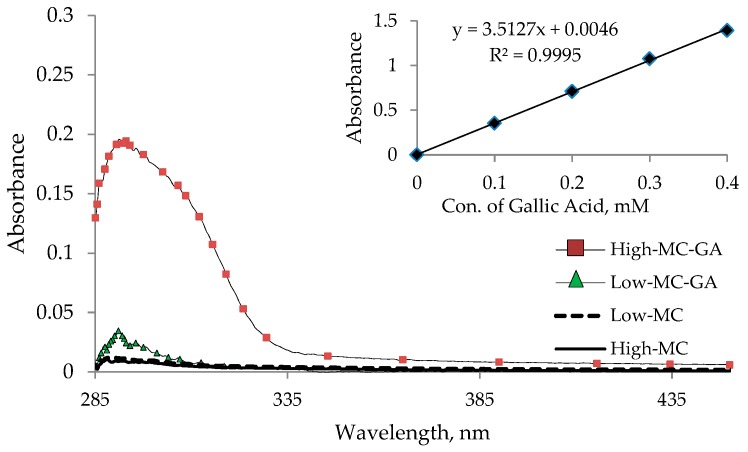
Absorbance spectra of the prepared methacrylamide chitosan-interpenetrating polymer network (IPN) hydrogels functionalized with gallic acid.

To quantify the amount of GA attached to the chitosan-IPN hydrogels, UV/Vis absorption measurements using a standard calibration curve based on GA were taken. The method relies only on the use of the free GA as the standard compound, and the results are given as moles of GA per surface area of hydrogel. Employing Beer’s Law regression at 293 nm, the quantities of GA per hydrogel were estimated to be ~0.019 μmol for low-MC-GA and 0.160 μmol for high-MC-GA (Figure 3), which were calculated from the total surface area of hydrogels with a size of 10.0 × 10.0 mm and a thickness of 0.24 mm. As illustrated in Table 1, a much higher quantity of attached antioxidant residues was found in the hydrogels with a longer MC polymer compared to those with a shorter MC. The longer chitosan seems to produce a more accessible site to GA for the amide coupling reaction as compared to its shorter counterpart, despite that the amounts of incorporated chitosans are nearly same regardless of the length of chitosans [16,34].

**Figure 4:**
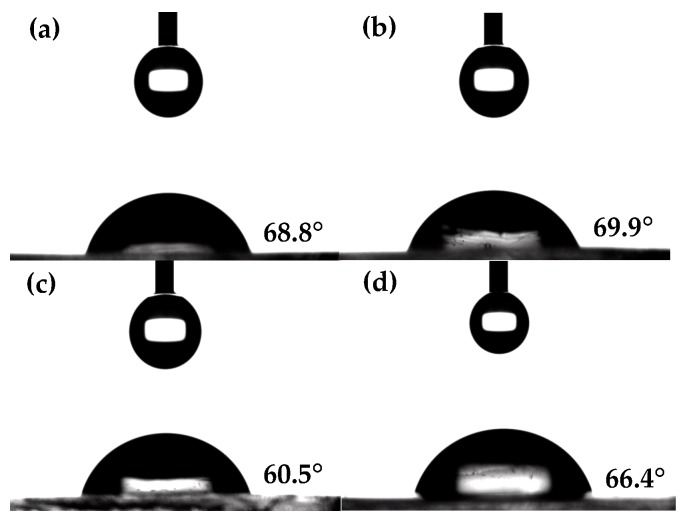
Contact angles of nanopure water droplets (4.5 μL) on (**a**) low-methacrylamide chitosan (MC) hydrogel; (**b**) low-MC-gallic acid (GA) hydrogel; (**c**) high-MC hydrogel; and (**d**) high-MC-GA hydrogel.

Contact angle measurements were carried out to investigate the surface properties of the prepared antioxidant hydrogels [39]. As shown in Figure 4 and Table 1, the surface modification of p(HEMA) hydrogels with cross-linked chitosan-IPN structures resulted in a decrease in the water contact angle, indicating enhanced surface wettability. Low- and high-MC-H exhibited contact angles of about 68.8° and 60.5°, respectively. These values represent decreases of about 4.4° and 12.7° for the hydrogels, respectively, relative to the value of 73.2° for the unmodified control. The observed decrease in the hydrogel contact angle was attributed to the relatively hydrophilic chitosan-IPN, which enhanced the surface-hydrophilicity of the prepared hydrogels. Moreover, the hydrogel surface-modified with longer chitosan networks showed higher wettability compared to its shorter counterpart, because a longer chitosan covered the hydrogel surface with its hydrophilic glucosamine units more so than shorter chains. However, the water contact angle of hydrogels functionalized with GA increased by about 1.1° and 5.9° for low- and high-MC-GA, respectively, relative to GA-unfunctionalized chitosan-IPN hydrogels, demonstrating that the relatively hydrophobic GA slightly decreased the surface wettability of the prepared hydrogels. Furthermore, the values depicted in Table 1 were in fairly good accordance with the contact angles reported in the literature [40]. Ketelson et al. have reported that commercially available contact lenses exhibited contact angles of 30–105° [40].

**Figure 5:**
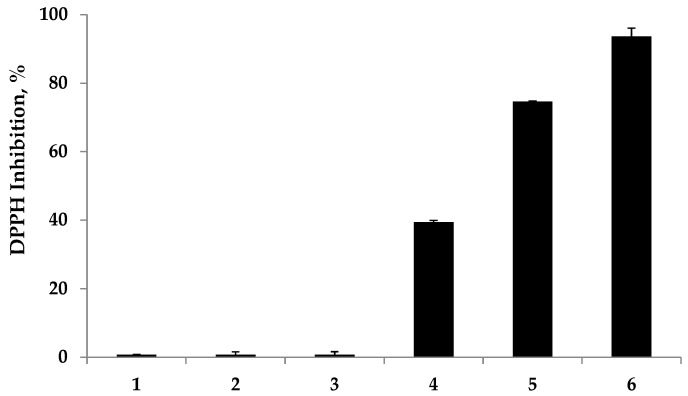
Radical scavenging capacity of the prepared antioxidant hydrogels against 2,2-diphenyl-1-picrylhydrazyl (DPPH) free radicals. The amount of ascorbic acid was 0.85 μmol. Legend: 1 = poly(2-hydroxyethyl methacrylate) (p(HEMA)); 2 = low-methacrylamide chitosan hydrogel (MC-H); 3 = high-MC-H; 4 = low-MC-gallic acid (GA); 5 = high-MC-GA; 6 = ascorbic acid.

**Figure 6:**
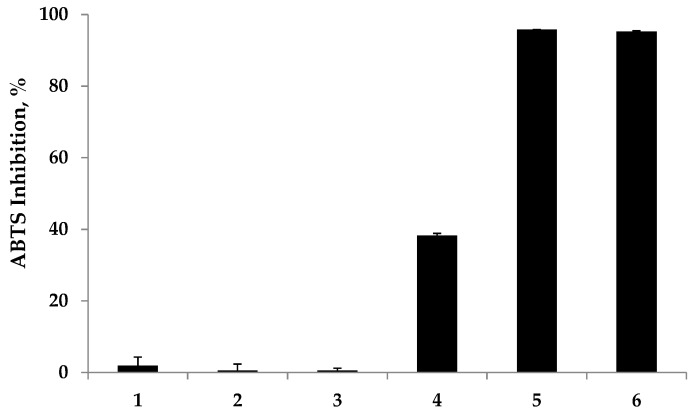
Radical scavenging capacity of the prepared antioxidant hydrogels against 3-ethylbenzothiazoline-6-sulfonic acid (ABTS) free radicals. The amount of ascorbic acid was 0.85 μmol. Legend: 1 = poly(2-hydroxyethyl methacrylate) (p(HEMA)); 2 = low-methacrylamide chitosan hydrogel (MC-H); 3 = high-MC-H; 4 = low-MC-gallic acid (GA); 5 = high-MC-GA; 6 = ascorbic acid.

The antioxidant properties of the fabricated chitosan-IPN hydrogels were assessed using DPPH and ABTS radical scavenging assays. Herein, the antioxidant efficiencies of the prepared hydrogels were investigated using ascorbic acid as a positive control. In the DPPH assay, the antioxidant activity is determined by the extent of the decolorization of the DPPH radical. The DPPH radical shows a strong absorption maximum at 517 nm and its color changes from purple to colorless followed by the formation of stable hydrazine (DPPH-H) upon the absorption of hydrogen from an antioxidant. Thus, the antioxidant effect is stoichiometrically proportional to the decrease in the UV absorption at 517 nm. In contrast, the ABTS assay is based on the reduction of the generated blue/green ABTS•+ species with the percent inhibition of the absorbance at 734 nm. As shown in Figure 5 and Figure 6, the radical scavenging abilities of the prepared hydrogels were evaluated upon reaction with DPPH and ABTS radicals. As expected, the polyphenol-free hydrogels did not exhibit any radical scavenging abilities. Notably, a remarkable improvement in the DPPH and ABTS radical scavenging abilities by the polyphenol-modified hydrogels was observed.

In the DPPH and ABTS assays, moderate antioxidant abilities were observed for low-MC-GA, which inhibited 39.40% and 38.25% of the DPPH and ABTS radicals, respectively. On the contrary, strong antioxidant activities were observed for the hydrogels with longer chitosan chains, which exhibited a 74.65% and 95.79% inhibition of DPPH and ABTS radicals, respectively, while the positive control, ascorbic acid, exhibited a 93.65% and 95.31% inhibition against DPPH and ABTS radicals, respectively.

The results suggest that hydrogels based on MC species with a higher MW exhibited stronger antioxidant effects than those with shorter MC moieties. This was attributed to the potent antioxidant residues being attached to the longer MC-based hydrogels. Generally, polyphenols possessing an *o*-diphenolic arrangement, for example, a catechol structure, can donate a hydrogen radical to scavenge DPPH and ABTS free radicals. The resulting phenolic radical is stabilized by resonance, whereby the radical is delocalized across the aromatic ring and is further oxidized to form the fully conjugated *o*-dione structure, *o*-quinone. Moreover, the additional hydroxyl group in GA enhances its antioxidant activity, as the added hydroxyl group adjacent to the *o*-dihydroxyl phenolic structure forms an intramolecular hydrogen bond in the *o*-position during the radical scavenging reaction, which provides additional stability to the phenoxy radical owing to its hydrogen-donating capacity. Several studies have reported the enhanced antioxidant activity of tri-hydroxyl derivatives in the *o*-position, such as catechin gallate ester and GA, because of the hydrogen-donating capacity of the third hydroxyl group to the phenoxy radical [24,41,42].

## 3. Materials and Methods

### 3.1. Chemicals

HEMA, EGDMA, 1-ethyl-3-(3-dimethylaminopropyl)-carbodiimide hydrochloride (EDC-HCl), N-hydroxysuccinimide (NHS), GA, APS, SMBS, methacrylic anhydride, DPPH, and ABTS were purchased from Sigma Aldrich (St. Louis, MO, USA). AIBN was purchased from Junsei (Tokyo, Japan), while chitosan (100–300 kDa and 600–800 kDa) was acquired from Acros Organics (Geel, Belgium). The degree of deacetylation was provided by the supplier and was found to be ≥90%. Deuterium oxide was purchased from Cambridge Isotope Laboratories (Tewksbury, MA, USA).

### 3.2. Synthesis of HEMA-Based Hydrogels

A HEMA monomer was initially purified using vacuum distillation prior to polymerization. Briefly, EGDMA (0.04 g) and AIBN (0.04 g) were dissolved in HEMA (9.92 g). The resulting solution was mixed for 30 min, injected into a square mold comprising two glass plates internally covered with a polypropylene sheet and separated by a 0.20 mm wide Teflon frame, and was heated at 90 °C for 5 h to allow for polymerization to take place. The samples were then removed from the molds and subjected to extensive dialysis. They were then placed in 400 mL of de-ionized water (changed three times daily), for 2 days, to remove any unreacted monomer and initiators. Subsequently, square hydrogels (10 mm × 10 mm × 0.24 mm) were cut from the square mold, immersed in boiling water for 15 min, and dried at 40 °C overnight.

### 3.3. Preparation of MC and Analysis of Degree of Methacrylation

MC was synthesized according to previously reported literature [43]. Chitosan of varying MW (*M*_w_ of 100,000–300,000 Da and 600,000–800,000 Da) was separately dissolved in 2 wt % acetic acid overnight at room temperature (RT) to constitute a 3 wt % solution of chitosan in distilled water. Methacrylic anhydride was added to the chitosan solutions at a 0.44 methacrylic anhydride/glucosamine molar ratio. The resulting mixture was stirred at RT for 3 h before being subjected to extensive dialysis against distilled water for 2 days with at least three to four changes of distilled water a day. The mixture was freeze-dried and stored at −20 °C until use. The degree of methacrylation of chitosan was determined using ^1^H-NMR spectroscopic measurements [35]. An appropriate amount of MC was dissolved in D_2_O to constitute a ~0.5% (*w*/*v*) MC solution. The degree of methacrylation was then calculated by comparing the integrated area of H2–H6 peaks at 2.8–4.0 ppm to that of the methylene peaks at 5.35 and 5.65 ppm. The ^1^H-NMR spectra were recorded using JNM-AL300 (JEOL, Tokyo, Japan).

### 3.4. Synthesis of MC-IPN Functionalized with GA

An appropriate amount of previously freeze-dried MC was dissolved to reconstitute a 2 wt % solution in distilled water. Then, previously prepared p(HEMA)-based hydrogels were immersed in the MC solution at RT. After 24 h, the p(HEMA)-based hydrogels were washed with distilled water and immersed in 10 mL of distilled water followed by the addition of polymerization initiators, APS and SMBS. The mixture was allowed to sit for 2 h to allow for the cross-linking reaction to proceed completely. The yield of the surface modification was calculated from Equation (1). To remove any unreacted cross-linking agents, MC-IPN hydrogels were washed with phosphate buffer saline (PBS; pH 7.4) for 3 days with at least four to five changes of buffer each day. Subsequently, the functionalization of MC-IPN hydrogels with GA was then performed. This was done by submerging the MC-IPN hydrogels in 20 mL of distilled water, followed by the addition of EDC-HCl, NHS and GA. The mixture was allowed to sit for 24 h at RT. The mixture was immersed in distilled water for 2 days to completely remove any unreacted chemicals prior to characterization.

% Yield = {(Weight of dried IPN Hydrogel − Weight of p(HEMA) hydrogel)/Weight of MC)} × 100(1)

### 3.5. UV-Vis Absorption Measurements

The absorption spectra of the hydrogels were measured at a wavelength range of 285–750 nm with a Shimadzu, UV-1650PC (Shimadzu, Tokyo, Japan) spectrophotometer. The measurements for each sample were repeated four times, and the results were averaged.

### 3.6. Contact Angle Measurements

A drop of nanopure water (4.5 μL) was positioned on the hydrogel surface. Contact angles were then measured using a DSA100 instrument (Krüss GmbH, Hamburg, Germany). The measurements for each sample were taken four times, and the results were averaged.

### 3.7. DPPH Radical-Scavenging Assay of the MC-IPN Hydrogels

A method described by Brand–Williams and modified by Miliauskas [44] was used in determining the DPPH radical-scavenging capacity of the prepared MC-IPN hydrogels. The test samples were compared to a known antioxidant, ascorbic acid (1000 ppm). Briefly, DPPH• solution (0.2 mM, in ethanol) was mixed with the hydrogel samples. The reaction mixture sample was shaken for 30 min at 37 °C in the dark. The reaction of the DPPH radical was estimated by measuring the absorption at 517 nm against ethanol as a blank in the spectrophotometer. The percentage of the DPPH• scavenging inhibition capacity was calculated from Equation (2):

% Inhibition = {1 − (Absorbance of sample/Absorbance of control)} × 100(2)

### 3.8. ABTS Radical-Scavenging Assay of the MC-IPN Hydrogels

The ABTS radical-scavenging capacity of each sample was determined according to the modified method described by Arnao et al. [45]. ABTS radical cations (ABTS•+) were produced by adding 7 mM ABTS solution and 2.4 mM potassium persulfate solution. The diluted ABTS•+ solution was then prepared by mixing the two solutions in equal quantities and allowing them to react for 24 h at RT in the dark. The solution was then diluted with methanol to obtain an absorbance range of 0.7–1 ± 0.02 units at 734 nm. Hydrogel samples were added to the diluted ABTS•+ solution and incubated for 30 min, at 37 °C, in the dark. The reaction of the ABTS•+ species was estimated by measuring the absorption at 734 nm against methanol as a blank. The percentage scavenging inhibition capacity of ABTS•+ was calculated using Equation (2).

## 4. Conclusions

We have prepared antioxidant p(HEMA)-based hydrogels using a chitosan-based IPN structure and surface immobilization with GA. We have successfully synthesized polymerizable MCs and applied them to the construction of chitosan-based IPN structures on p(HEMA) hydrogels. Remarkably, the IPN synthesis was carried out in an aqueous solution without an additional cross-linker, which makes this approach more facile and practical than those previously reported using chitosan-based IPN structures. Further covalent modifications with GA on the chitosan backbone yielded antioxidant chitosan-IPN hydrogels. Superior antioxidant effects were observed by the hydrogels with longer chitosan species, as more antioxidant residues were attached to the longer chitosan chains. The surface wettability of the prepared antioxidant hydrogels was enhanced in the presence of the relatively hydrophilic chitosan-IPN structure but was slightly decreased upon conjugation with GA. The results described herein support the feasibility of chitosan-IPN hydrogels as versatile platforms for the development of ophthalmic materials and functional biomaterials with intrinsic bioactivities and biocompatibility.

## Figures and Tables

**Table 1:** Characteristics of prepared antioxidant hydrogels.

Hydrogels	MW of Chitosan (kDa)	Amounts of Attached Polyphenols per Hydrogel (μmol) ^a^	Contact Angle (°) ^b^
p(HEMA) ^c^	—	—	73.2 ± 1.9
Low-MC-H	100–300	—	68.8 ± 2.9
High-MC-H	600–800	—	60.5 ± 12.3
Low-MC-GA	100–300	0.019 ± 0.0028	69.9 ± 4.1
High-MC-GA	600–800	0.160 ± 0.0536	66.4 ± 5.0

^a^ Data are means ± SD (*n* = 3); ^b^ Data are means ± SD (*n* = 4), ^c^ p(HEMA) is pristine p(HEMA)-based hydrogel.
